# Cell Therapy for Colorectal Cancer: The Promise of Chimeric Antigen Receptor (CAR)-T Cells

**DOI:** 10.3390/ijms222111781

**Published:** 2021-10-29

**Authors:** Cristina Aparicio, Marina Belver, Lucía Enríquez, Francisco Espeso, Lucía Núñez, Ana Sánchez, Miguel Ángel de la Fuente, Margarita González-Vallinas

**Affiliations:** Unidad de Excelencia Instituto de Biología y Genética Molecular (IBGM), University of Valladolid (UVa)-CSIC, 47003 Valladolid, Spain; cristina.aparicio@uva.es (C.A.); marina.belver@uva.es (M.B.); luciaen96@gmail.com (L.E.); ecfran7@gmail.com (F.E.); nunezl@ibgm.uva.es (L.N.); asanchez@ibgm.uva.es (A.S.); mafuente@ibgm.uva.es (M.Á.d.l.F.)

**Keywords:** colorectal cancer, cell therapy, chimeric antigen receptor (CAR), CAR-T cells, immunotherapy, tumour-associated antigen (TAA), tumour-specific antigen (TSA), neoantigens, preclinical development, clinical trials

## Abstract

Colorectal cancer (CRC) is a global public health problem as it is the third most prevalent and the second most lethal cancer worldwide. Major efforts are underway to understand its molecular pathways as well as to define the tumour-associated antigens (TAAs) and tumour-specific antigens (TSAs) or neoantigens, in order to develop an effective treatment. Cell therapies are currently gaining importance, and more specifically chimeric antigen receptor (CAR)-T cell therapy, in which genetically modified T cells are redirected against the tumour antigen of interest. This immunotherapy has emerged as one of the most promising advances in cancer treatment, having successfully demonstrated its efficacy in haematological malignancies. However, in solid tumours, such as colon cancer, it is proving difficult to achieve the same results due to the shortage of TSAs, on-target off-tumour effects, low CAR-T cell infiltration and the immunosuppressive microenvironment. To address these challenges in CRC, new approaches are proposed, including combined therapies, the regional administration of CAR-T cells and more complex CAR structures, among others. This review comprehensively summarises the current landscape of CAR-T cell therapy in CRC from the potential tumour targets to the preclinical studies and clinical trials, as well as the limitations and future perspectives of this novel antitumour strategy.

## 1. Introduction

As life expectancy grows in the 21st century, cancer diseases have been shown to be one of the main causes of death, and one of the main obstacles for this life expectancy to keep on growing [1]. In 2020, there were approximately 19 million new cancer cases and almost 10 million cancer deaths worldwide, colorectal cancer being the third most diagnosed type of tumour (10.0% of all new cancer cases) and the second most lethal (9.4% of all cancer deaths) [2].

The molecular features of cancer, known as “cancer hallmarks”, include the accumulation of a variable number of genetic mutations and the loss of normal cellular regulatory processes. This leads to the expression of several antigens that may allow the immune system to distinguish a cancerous cell from their normal counterpart [3,4]. The recognition of these antigens implies the activation of an antitumour response; however, tumour cells are frequently able to evade the immune response in various ways, thus allowing cancer development and spread even in immunocompetent organisms [5]. In the particular case of colorectal cancer (CRC), it has its origin in aberrant crypt foci which may evolve to adenoma and later to invasive carcinoma. The genomic changes responsible for this progression to colorectal cancer are mainly caused by genomic instability due to three major mechanisms: chromosomal instability, microsatellite instability and the CpG island methylator phenotype [6]. Commonly, genomic changes related to colorectal tumorigenesis include a loss of at least one wild-type copy of a tumour-suppressor gene such as *APC*, *P53* or *SMAD4* [7].

CRC presents high rates of metastasis, since up to 50% of the patients develop metastasis at some point of the disease. In this context, molecular factors have arisen as biomarkers that contribute to estimate the progression of the disease (prognostic biomarkers) or to predict the response to cancer therapies (predictive biomarkers). For example, activating RAS (KRAS/NRAS) mutations are commonly used as predictive biomarkers, since they are associated with resistance to anti-epidermal growth factor receptor (EGFR) antibodies [8]. Recently, thanks to the improved knowledge of the molecular pathways involved in CRC tumorigenesis, a Consensus Molecular Subtype (CMS) classification has been proposed, which is based on the molecular profile of the tumour and contributes to the determination of patient prognosis in combination with the TNM staging system [6].

Colorectal cancer treatment should always contemplate surgical resection of the tumour, alone or in combination with chemotherapy and radiotherapy, as it has shown to be the most effective way of increasing patient survival. However, surgery is not useful for CRC diagnosed at advanced stages, which represents approximately 25% of cases [9]. Pharmacological approaches include non-specific drugs such as fluoropyrimidines (5-fluorouracil, usually combined with leucovorin, or capecitabine, a 5-fluorouracil prodrug), irinotecan and oxiplatin [10], as well as targeted drugs such as angiogenesis inhibitors (e.g., bevacizumab), EGFR inhibitors (e.g., cetuximab) and multikinase inhibitors (e.g., regorafenib) [6,11]. Combinational regimes and targeted drugs have substantially improved CRC treatment over the last years, especially of patients with metastatic disease. However, the 5-year overall survival (OS) rate of CRC patients in the United States is estimated at 64% for all stages and at only 12% for metastatic CRC (mCRC) [6], pointing to the urgency of developing new therapeutic strategies for this disease.

Novel medicinal products such as cell therapy, and especially chimeric antigen receptor (CAR)-T cells, have emerged as promising therapeutic tools for cancer treatment. The remarkable success of this kind of treatment in advanced haematological malignancies has led to numerous studies to assess their utility in solid tumours. In this review, we aim to summarise the current knowledge and preclinical and clinical studies of CAR-T cell therapies in CRC treatment, as well as to discuss the limitations and future work needed to make this new therapeutic tool widely available in the clinical setting.

## 2. Cell Therapy Strategies for CRC Treatment

Most anticancer cell therapies are based on the use of immune cells to fight the disease, thus belonging to the group of the immunotherapeutic approaches. Cancer immunotherapy includes several strategies, such as the administration of stimulating cytokines, monoclonal antibodies targeting immune checkpoint inhibitors, vaccines based on dendritic cells (DC), etc. Among them, some of the most promising strategies are based on the use of T cells directed to the tumour, including tumour-infiltrating lymphocytes (TILs) and T cells genetically modified to express receptors specifically designed to target the tumour (TCRs and CARs) [12,13]. Besides these strategies based on T cells, natural killer (NK) cell therapy has also been assayed against cancer with promising results [14,15,16]. Below, we describe the most common cell therapeutic approaches directed concretely against CRC.

The cytokine-induced killer (CIK) cells, a frequently studied cell immunotherapy in CRC, are a heterogeneous group of cells obtained from peripheral blood mononuclear cells (PBMCs) stimulated ex vivo with an anti-CD3 antibody and a cocktail of cytokines [17,18,19]. These cells share functional and phenotypic properties with NK and T cells and are characterised by rapid expansion ex vivo, non-major histocompatibility complex (MHC)-restricted tumour-killing activity, strong antitumour activity and minimal toxicity [17,18]. It has been observed that the combination of adjuvant chemotherapy with sequential infusions of CIK cells significantly improved the progression-free survival (PFS), disease-free survival (DFS) and OS rates of CRC patients [18,19], especially in those with high-risk T4 stage and insufficient duration of chemotherapy (DFS and OS) [18]. Moreover, this combination therapy has also been used on mCRC patients, showing good tolerability and a significant increase in OS [20].

On the other hand, a different kind of cell immunotherapy is the DC vaccine, which is based on the stimulation of the adherent PBMCs with the lysate of specific tumour cells and other stimulating factors. This approach has been used in combination with CIK therapy, obtaining an increase in quality of life (QOL) [21,22], no toxic effects and a significantly longer OS [21,22,23], DFS [23] and PFS [21], even in stage III/IV CRC patients [21]. Additionally, Ishii et al. combined this immunotherapy with interferon (IFN)-α therapy (IFN-α gene-transduced tumour cells) showing growth inhibition of established tumours in mouse models [24].

Several studies utilise the combination of natural killer (NK) therapy with cetuximab, a first-line treatment for EGFR-positive mCRC that also interacts with NK cells, triggering antibody-dependent cell-mediated cytotoxicity (ADCC). It has been demonstrated that activated peripheral blood NK cells are cytotoxic for CRC tumour cells but, in combination with cetuximab, it potentiates the antitumour activity on EGFR-positive CRC cell lines (either RAS^wt^, RAS^mut^ or BRAF^mut^) [25] and in EGFR-positive CRC xenograft models [26]. Furthermore, the umbilical cord blood stem-cell-derived NK (UCB-NK) cells have a better cytotoxic effect than peripheral blood NK cells in vitro and, in mouse models, UCB-NK cells significantly increase the life span with respect to the cetuximab [27]. Li et al. evaluated the administration of autologous NK cell therapy in combination with conventional chemotherapy in a clinical trial, showing a significant increase in PFS and OS in advanced colon carcinoma patients, especially with poorly differentiated carcinomas [28].

A successful strategy based on T cells was the combination therapy of XELOX (capecitabine and oxaliplatin) and bevacizumab together with αβ T cells, previously expanded ex vivo by anti-CD3 and IL-2 stimulation. The treatment achieved an 80% response rate and acceptable toxicity in stage IV CRC patients [29]. Another T-cell-based strategy is TCR therapy, which is based on genetically modifying T cells to express transgenic TCRs capable of recognising specific tumour antigens. Parkhurst et al. developed a transgenic TCR that specifically bound CEA-positive CRC cells, leading to an enhancement in tumour recognition in comparison with the wild-type T cells [30].

### CAR-T Cell Therapy

A strategy that is showing a strong efficacy in terms of antitumour activity is CAR-T cell therapy. Born in 1989 [31] as a way to direct the specificity of T cells in a MHC-unrestricted manner, it has been recently applied to enhance the immune system’s response to cancer cells when the natural response has failed [32]. CAR-T cells are T cells genetically modified to express engineered receptors, chimeric constructs, that recognise cancerous antigens in an MHC-independent manner, and are able to react specifically against them [33,34,35].

Different strategies have been used to induce CAR expression in T cells. Viral transductions, using both ɣ-retroviral and lentiviral vectors, are the most common approaches. Other integrating methods are performed with the use of transposons such as Sleeping Beauty or PiggyBac transposon systems. Non-integrating non-viral approaches have also been studied, the most common being the electroporation of CAR-encoding mRNA. Moreover, additional strategies, such as the use of DNA minicircles or the combination of Cas9 RNP together with AAV6 as CAR donors, have also been developed [34].

**Figure 1:**
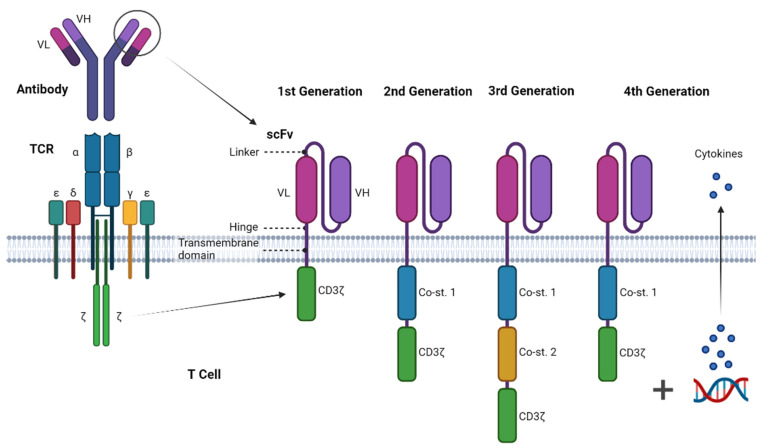
Structure of chimeric antigen receptors (CARs) and the different CAR generations. The first CAR generation is composed of a single-chain variable fragment (scFv) followed by a hinge, a transmembrane domain and an intracellular region, most commonly TCR signalling component CD3ζ. The second and third CAR generations include one or two co-stimulatory domains (Co-st.), respectively, being usually derived from CD28 and 4-1BB receptors, among others. Fourth-generation CARs or TRUCKs usually combine a second-generation CAR construct with the constitutive or inducible expression of chemokines. Created with Biorender.com.

CAR receptors consist of a target-binding extracellular region that confers antigen specificity, commonly based on a single-chain fragment of variable region antibody (scFv), a hinge and transmembrane region, and an intracellular domain that mediates T-cell activation, mainly via the TCR CD3ζ signalling chain (Figure 1). In this intracellular domain, other types of primary stimulation signalling chains have been used, such as the FcεRIγ-chain, CD3ε, DNAX-activation protein 10 (DAP10), ZAP70, lck and fyn, being the first-generation CARs [36]. The second-generation CARs include one co-stimulatory domain in the intracellular region such as CD28, 4-1BB (CD137), OX40 (CD134), CD27 or ICOS. These domains increase the proliferation, cytotoxicity, sustained response and persistence in vivo. In the third generation, CAR receptors contain two co-stimulatory domains in order to increase the cytokine production and antitumour effect [31]. Furthermore, there is a fourth generation, also called T cells Redirected for Universal Cytokine Killing (TRUCKs), which combine the CAR with the increased secretion of cytokines (e.g., IL-2, IL-12) [37]. This generation is able to increase T cell activation, recruit and activate endogenous innate immune cells to the tumour, modify the tumour microenvironment and increase the T cell persistence [31].

Currently, multiple strategies are being developed to further improve CAR-T cell therapies, such as those directed to confer them the ability to inhibit the tumour immunosuppressive microenvironment by eliminating programmed cell death 1 (PD-1) and/or cytotoxic T lymphocyte antigen 4 (CTLA-4), or to improve specificity and safety by CARs targeting two different tumour antigens, among others. Moreover, many efforts are focused on the development of genetic modification strategies to make CAR-T cells from healthy donors a therapy suitable for allogeneic use [33].

**Figure 2:**
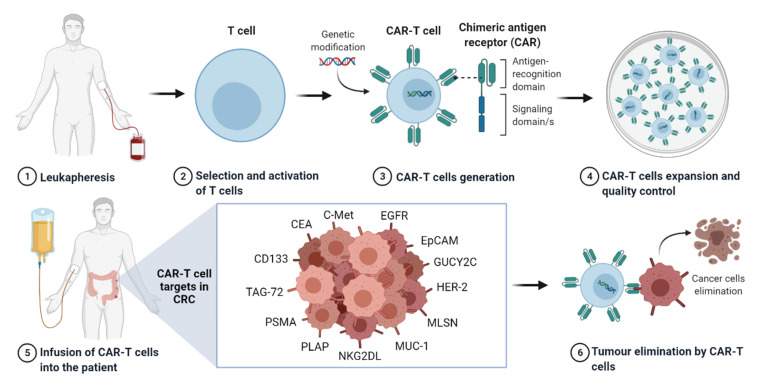
CAR-T cell therapy approach against colorectal cancer. Firstly, human T cells are obtained, selected and activated in vitro. Genetic modification is performed to induce CAR expression in the T cell, and the obtained CAR-T cells are expanded and formulated to manufacture the CAR-T cell product with the corresponding quality controls. The CAR-T cell product is administered to the patient, where it is expected to eliminate the tumour cells by targeting the different CRC antigens. Created with Biorender.com.

Over the years, CAR receptors have evolved to more effective and safer variations, being able to recognise a wide repertoire of antigens in both haematological and solid malignancies [32]. Since the birth of this type of cell therapy, we have witnessed remarkable achievements in the field, with a total of 988 clinical trials reported by 2021 [38]. To date, there are already three CAR-T cell therapies (axicabtagene ciloleucel, tisagenlecleucel and brexucabtagene autoleucel) approved for commercialisation by the Food and Drug Administration (FDA) and the European Medicines Agency (EMA) for the treatment of leukaemia and lymphomas, all targeting CD19-positive haematological malignancies. Moreover, numerous preclinical and clinical studies are testing CAR-T cell strategies to treat solid tumours [39]. Regarding CRC, several CAR-T cell approaches have been currently developed to target different tumour antigens (Figure 2). The current advances in the field of CAR-T cell therapy for CRC are described and discussed in the following sections.

## 3. Molecular Targets of CAR-T Cells against CRC

A critical issue in the development of CAR-T cell therapies for cancer treatment is the identification of target antigens specific for each cancer type. The selection of the target/s will influence specificity, efficacy and toxicity, thus determining therapy success. Cancer-produced antigens are classified as tumour-associated antigens (TAAs) and tumour-specific antigens (TSAs), also called neoantigens [40]. The former are antigens expressed in normal cells but overexpressed in tumour cells. These TAAs are easy to identify and are shared by many patients, but targeting them with CAR-T cells may cause severe adverse effects due to their low specificity. As TAAs, in CRC we can find the carcinoembryonic antigen (CEA), mucin-1 (MUC-1), survivin, Wilms tumor-1, ARG1, ST4, and MYB, among others [40,41]. Additionally, there are cancer/testis antigens (CTAs), which are normally only expressed in the testicles and are immunogenic and overexpressed in CRC, such as melanoma-associated antigen (MAGE)-A, PASD1, NY-ESO-1, LAGE-1, OIP5, TTK, PLU1, DKKL1 and FBXO39 [41,42].

TSAs are dysfunctional peptides derived from the expression of non-synonymous somatic mutations (insertions/deletions, single-nucleotide variations, frameshift mutations, structural variations and fusion genes), thus being only expressed in tumour cells [42]. This specificity provides TSAs with a high value as therapeutic targets due to the low probability of on-target off-tumour effects. Although some peptides derived from mutated *TGFΒRII, RNF43, UBR5, XYLT2, DPAGT1, REPIN1, BRAF, TP53, RNF213*, *TUBGCP2* and *KRAS* genes are immunogenic and could be promising targets for CAR-T cells, most currently studied CAR-T cells are directed towards TAAs, mainly due to the low frequency of those specific mutations among CRC patients [41,42]. The different antigens targeted by the CAR-T cell therapies developed for CRC and assayed in preclinical studies and clinical trials to date are summarised below.

**CEA**, also known as CEACAM5, is a foetal glycosylphosphatidylinositol-anchored glycoprotein belonging to the family of immunoglobulins. It is expressed at an early stage of human embryo and foetal development but is almost undetectable in normal adult tissues, except in the gastrointestinal tract at a low level [43,44]. It is overexpressed in many tumours, such as colon, pancreatic, gastric, lung and ovarian cancers. Furthermore, in cancer, the apical polarity of the CEA is lost and it enters the capillaries, so it increases in blood serum by around 43% [43,45]. Although its function and signalling is yet unclear, CEA is overexpressed in 98.8% of CRC tissues, so it is a useful diagnostic and prognostic tumour marker, as well as a promising target for novel treatments against CRC [43,44].

Guanylyl cyclase 2 C (**GUCY2C**) belongs to a family of mucosal cyclase receptors and is only expressed in intestinal epithelial cells from the duodenum to rectum, with the exception of a subset of hypothalamic neurons [46,47,48]. This receptor catalyses the conversion of GTP into cyclic GMP, triggering the activation of cGMP-related signalling pathways which regulate intestinal homeostatic processes such as epithelial cell proliferation, differentiation and apoptosis [46]. The dysregulation of this signalling axis promotes the appearance of inflammatory bowel disease and bowel transit disorder, as well as cancer [46]. In the early stages of tumour initiation, loss of the GUCY2C-binding ligands (guanylin or uroguanylin) occurs, resulting in silencing of the signalling pathway and promoting intestinal transformation [46,48]. GUCY2C is overexpressed in nearly 95% of CRCs, including mCRC, as well as in pancreatic and a subset of gastroesophageal tumours [46].

Natural killer group 2 member D (NKG2D) is a C-type lectin-like receptor characteristically expressed on NK cells, and also in CD8^+^ T cells, γδ T cells and some autoreactive or immunosuppressive CD4^+^ T cells [49]. This receptor activates immune cells through the adaptor molecule DAP10, triggering proliferation, pro-inflammatory cytokine production (IFN-γ, IL-2) and cytotoxic functions [49,50]. It has eight different ligands (**NKG2DL**) belonging to the cytomegalovirus UL16-binding proteins (ULBP1-6) and MHC-I Chain-related molecules (MIC-A and B) families in humans [49,51]. These ligands are expressed in response to external signals such as stress or pathogens and during neoplastic cell transformation but they are rarely present in healthy tissue [49,50]. Additionally, they can be present in soluble form, impairing NKG2D-dependent functions, and they are associated with poor clinical prognosis and metastasis in high levels [51]. NKG2DL are expressed in a variety of tumours, including carcinomas (ovarian, colon, cervical, breast, lung, hepatocellular, renal, prostate, pancreatic and head-and-neck cancers), leukaemia, lymphoma, multiple myeloma, melanoma, glioma, osteosarcoma and neuroblastoma [51].

**EGFR** is a transmembrane glycoprotein, belonging to the protein kinase superfamily [52]. Ligand binding induces receptor dimerization, triggering cell proliferation, survival and differentiation. Recently, new EGFR functions related to autophagy and metabolism, which are induced by cellular and environmental stresses and activated in cancer cells, have been characterised [53]. This receptor is frequently overexpressed and/or mutated in most solid tumours, including CRC, glioblastoma and breast, renal, ovarian, head-and-neck, brain and non-small-cell lung cancers [52,53]. Some immunotherapy targets are specific oncogenic mutations such as EGFR variant III (**EGFRvIII**), which seems to be exclusively expressed in tumour tissue [33,54]. This mutation is located in the extracellular domain of EGFR and triggers ligand-independent activation [53].

Another member of the EGFR family is the human epidermal growth factor receptor 2 (**HER-2**) which does not bind ligands, being activated by heterodimerization with a ligand-bound receptor of its family [55]. HER-2 often dimerises with HER-3, triggering proliferation, angiogenesis and metastases. HER-2 is overexpressed in oesophageal adenocarcinoma and breast, gastric, lung, pancreatic and colorectal cancers (rates from 2% to 11% in 2% of all CRCs) [55,56]. Recent studies have shown that both HER-2 and HER-3 are overexpressed in liver metastasis CRC patients (8% and 75%, respectively), so they are both promising therapeutic targets for novel treatments [56].

The epithelial cell adhesion molecule (**EpCAM**) is a type I transmembrane glycoprotein expressed mostly in the basolateral membrane of normal epithelial cells [57]. In normal conditions, EpCAM is involved in cell–cell adhesion and regulates the differentiation in progenitor and embryonic stem cells; however, in the context of cancer, its overexpression is related to increased cell proliferation, migration, invasion and tumour metastasis [57,58]. Although it is overexpressed in a wide variety of epithelial tumours, it is associated with poor prognosis only in some cancer types (colorectal, breast, prostate, gallbladder, ovarian, bladder, pancreas and adenoid cystic carcinomas) while it is reported to be a marker of good prognosis in other tumours (oesophageal, renal, gastric, endometrial, thyroid and head-and-neck carcinomas) [59]. In CRC, it is overexpressed in more than 90% of all cancer cells [58]

The tumour-associated glycoprotein (TAG)-72 is a membrane-bound glycoprotein that is not expressed in most normal tissues, except for the endometrium during the secretory phase and the foetal tissue. **TAG-72** is overexpressed in 80% of CRCs in comparison with the normal mucosa, and it is elevated by 43% in the blood serum of CRC patients [45,60]. This glycoprotein is also overexpressed in breast, oesophagus, stomach, pancreas, ovarian and lung cancers, being associated with poor prognosis [60].

Mesothelin (**MSLN**) is a glycosylphosphatidylinositol-anchored protein which is involved in several mechanisms of cancer pathogenesis such as increased cell proliferation and survival, but its biological functions remain uncertain [61]. Normally, its expression in humans is limited to the mesothelial cells of the pericardium, pleura, peritoneum and tunica vaginalis [61]. In relation to cancer, MSLN is overexpressed not only in CRC, but also in pancreatic, ovarian, lung, gastric, cervical, endometrial and biliary cancers, malignant pleural mesothelioma, uterine serous carcinoma, cholangiocarcinoma and paediatric acute myeloid leukaemia [61,62]. With regard to CRC, up to 60% of the malignancies have been found to be positive for MSLN expression [62].

Mucin-1 (**MUC-1**) is a transmembrane mucin expressed in the apical surface of secretory epithelial cells and in hematopoietic cells [41]. It is overexpressed in several cancers, such as colorectal, lung, pancreatic, prostate, ovarian, lung, breast and prostate cancers, where it loses its apicality [63].

Placental alkaline phosphatase (**PLAP**) is a metalloenzyme that catalyses the hydrolysis of phosphoric acid monoesters and is mainly expressed in placenta, but it is also present at low levels in uterine cervix, fallopian tube, testis and lung [64,65]. PLAP is overexpressed in CRC and it is detected in more than 20% of colorectal adenocarcinomas. Additionally, it is overexpressed in malignant seminomas, teratomas and ovarian and cervical carcinomas [64].

Cluster of differentiation 133 (**CD133**) or prominin-1, is a five-transmembrane glycoprotein located in the membrane protrusions. It is expressed in several progenitor and stem cells and it is involved in the organization of the topology of the plasma membrane [66]. CD133 has been proposed as a cell surface marker of ovarian, pancreas, lung, colorectal, brain and kidney cancer stem cells. In CRC, CD133 expression is related to chemoresistance, a higher rate of distant metastasis and metastatic relapse [66,67].

Mesenchymal–epithelial transition factor (**c-Met**) is a tyrosine kinase receptor expressed in cells of epithelial–endothelial origin, including liver cells, fibroblasts, haematopoietic cells and keratinocytes [68]. It is related to cell proliferation, motility, tissue regeneration, wound healing, epithelial to mesenchymal transition and angiogenesis [68,69]. c-Met is overexpressed in colorectal, lung, pancreatic, gastric, head-and-neck, ovarian, renal, prostate and breast cancers [68]. In CRC, c-Met is overexpressed in about 15% of the patients [68] and is involved in cell growth, differentiation, cancer progression and metastasis [68,69].

Prostate-specific membrane antigen (**PSMA**), also known as folate hydrolase I or glutamate carboxypeptidase II, is a transmembrane protein characteristic of the membrane of prostate epithelial cells but also expressed in salivary glands, duodenal mucosa, a subset of proximal renal tubular cells and a subpopulation of neuroendocrine cells in the colonic crypts. In cancer, PMSA is overexpressed not only in prostate epithelial tumour cells, but also in subtypes of transitional cell carcinoma, renal cell carcinoma, colon carcinoma and the peritumoral and endotumoral endothelial cells of the neovasculature [70]. In CRC, PSMA is expressed in about 75–80% of primary tumours and metastases [71].

## 4. Preclinical Studies on CAR-T Cells Directed against CRC

The preclinical studies related to the development of novel CAR-T cells for CRC treatment are summarised in Table 1 (in vitro results) and Table 2 (in vivo results). One of the first preclinical studies in this regard was published in 2017 by Ang et al., and focused on the cytotoxic effect of EpCAM-directed CAR-T cells. The study showed that multiple repeated infusions of anti-EpCAM CAR-T cells, generated by mRNA electroporation, delayed disease progression in immunodeficient mice bearing CRC xenografts [72]. Together with EpCAM, CEA is one of the most studied targets for anti-CRC CAR-T cells. Furthermore, second-generation CAR-T cells targeting CEA have demonstrated excellent antitumor results both in vitro and in vivo, that are increased when combined with interleukins such as IL-12 [73].

The cytotoxic effects of CAR-T cells targeting different CRC antigens have been demonstrated against a wide variety of human CRC cell lines, including Caco-2, DLD-1, HRT-18G, HT-29, LoVo, LS-C, LS123, LS174T, SW480, SW620 and T84, and some of them have also been used to obtain the mouse xenografts for in vivo experiments (Table 1 and Table 2). The fact that each cell line expresses different antigens with distinct antigenic density will influence the results of the antitumour efficacy obtained for a specific type of CAR-T cell, as outlined by Zhang et al. [74].

Regarding the genetic modification strategy to manufacture the novel CAR-T cells against CRC, those based on viral vectors (both ɣ-retrovirus and lentivirus) are the most commonly used approach, mostly due to their high efficiency [31]. Moreover, non-viral approaches are also assayed, but to a lesser extent. One example is the study of Ang et al. previously mentioned, in which they produced anti-EpCAM CAR-T cells by mRNA CAR electroporation. In a different study, Deng et al. electroporated T cells in the presence of a NKG2D-CAR minicircle DNA vector, obtaining a CAR-T cell product with more than 70% CAR+ cells; however, electroporation importantly reduced cell viability and CAR expression decreased in a time-dependent manner [75]. These approaches, in which the CAR is transiently expressed, have the advantage of having less toxic effects, but the persistence and potency of CAR-T cells is reduced, thus requiring re-inoculation to achieve the same antitumour effects as with the stably modified CAR-T cells [72].

Both second- and third-generation CAR-T cells are used for in vitro and in vivo studies. In all of them, the co-stimulatory domains used are CD28 and/or 4-1BB. Although Ramos et al. demonstrated that third-generation CD19-CAR-T cells in patients with non-Hodgkin’s lymphomas had better expansion and persistence than second-generation [76], there is no direct comparison between different generations of CAR-T cells in the context of CRC. Tandem CAR-T cells targeting both CEA and CD30 showed significantly increased cytotoxicity, persistence and release of perforin and granzyme B in comparison with CEA-CAR-T cells. Similarly, CD30/TAG72-CAR-T also showed increased cytotoxicity in comparison with TAG72-CAR-T cells. On the contrary, a tandem CAR-T cell against CEA and CD25 showed increased cell persistence but similar cytotoxicity against CRC models in comparison with anti-CEA-CAR-T cells [77].

CAR-T cell doses for in vivo studies in CRC mouse models usually range from 2 × 10^6^ [78] to 2 × 10^7^ [74]. In the xenograft models, CAR-T cells are inoculated either together with tumour cells or independently once the tumour is established, a few days after tumour cell administration. Several studies using CAR-T cells targeting CEA or EpCAM showed that co-inoculation of CAR-T and tumour cells inhibited or delayed tumour formation [75,76,77]. Furthermore, when CEA-CAR-T cells were co-inoculated with IL-7- and IL-12-expressing mesenchymal stem cells, tumour inhibition was enhanced, resulting in prolonged survival [79]. On the other hand, when the cells are administered once the tumour is formed, several CAR-T cell doses are usually administered. Among these studies, Huang et al. reported a complete eradication of the tumour with the use of EGFRvIII-CAR-T cells in combination with miR-153, which inhibits indoleamine 2,3-dioxygenase 1 (IDO1), inversely associated with patient survival [54]. Additionally, the combination of CEA-CAR-T cells with rhIL-12 provides a better antitumour effect than CAR-T cells alone [73]. These results suggest that the combination with cytokines or other therapeutic approaches can enhance CAR-T cell antitumour activity, as observed in CRC animal models. However, CAR-T cell therapy alone against the CRC targets CEA [73], EpCAM [72], GUCY2C [48], NKG2DL [75] and PLAP [80] have also demonstrated antitumour efficacy in preclinical studies. Although CAR-T cells are usually administered intravenously, intraperitoneal infusion has also been assayed with CAR-T cells targeting EpCAM [72], and GUCY2C [48], in order to increase efficacy and safety through local administration [81].

Regarding patient-derived-xenograft (PDX) mouse models of CRC, which have been shown to reflect the clinical and molecular heterogeneity of the patients and to recreate the tumour-immunosuppressive microenvironment [78,82,83], have also been used in two preclinical studies of anti-CRC CAR-T cells. One of them showed significant antitumour efficacy after administration of a third-generation MSLN-CAR-T and the persistence of the cells a minimum of 10 days after their administration [82]. The other study demonstrated that HER2-CAR-T cells were able to induce tumour regression or even elimination in a PDX mouse model of CRC and protected the treated animals from tumour rechallenge [78].

Due to the promising results obtained in preclinical studies, many of the CAR-T cell therapies developed against CRC are being already evaluated for antitumour efficacy and safety in clinical trials.

**Table 1:** In vitro studies of CAR-T cell cytotoxicity against human colorectal cancer cell lines.

Target	Gen.	Co-st.	Vector	Cell Line	Ratio (Effector: Target)	Results	Ref.
CD133	2nd	4-1BB	LV	SW620	1:1; 5:1	Significant elimination of target cells (% C (5:1): ~40%).	[84,85]
CEA	2nd	4-1BB	LV	HT29	4:1; 2:1; 1:1; 1:2; 1:4	% C (2:1): ~75%, that significantly increases with rhIL-12 (% C (2:1): ~90%) and releases a higher concentration of IL-2 and IFN-γ.	[73]
	2nd	CD28	RV	LS174T	1:2:0.02 (MSC)	% C: ~60%, significantly increased in combination with IL7/IL12 expressing MSCs (% C: ~80%).	[79]
CEA CD30/CEA CD25/CEA	2nd	CD28	RV	LS174T	3:1; 2:1; 1:2; 1:5	CD30/CEA-CAR-T cells induce higher cytotoxicity (% C (1:2): ~70%) than CEA-CAR-T and CD30-CAR-T. CD25/CEA-CAR-T has similar cytotoxic effects to CEA-CAR-T (% C (1:5): ~15%). Only CD30/CEA CAR-T enhanced the release of perforin and especially granzyme B.	[77]
EGFRvIII	3rd	CD28 4-1BB	LV	DLD-1, HCT116	30:1; 10:1; 3:1; 1:1	% C (10:1): ~80% DLD-1 and 65% HCT116, and increase in caspase 3/7 proteins release. Combination with miR-153 (that inhibits IDO1 expression) enhances CAR-T antitumor activity.	[54]
EpCAM	3rd	CD28 4-1BB	LV	SW480, HT29	2.5:1; 5:1; 10:1	% C (10:1): ~50% SW480 and HT29. ↑ Release of IFN-γ and TNF-α.	[74]
	2nd	4-1BB	LV	SW620, SW480, HCT116, HT29, LoVo,	0.5:1, 1:1, 2:1, 4:1, 8:1;16:1	% C (16:1): ~60% SW620, 55% SW480, 50% HCT116, 40% LoVo and HT29. ↑ Release of IFN-γ, IL-2 and IL-6.	[86]
	3rd	CD28 4-1BB	mRNA	HRT-18G	1:1; 2.5:1; 5:1; 10:1; 20:1	% C (10:1): ~45%. ↑ Release of IFN-γ and granzyme B.	[72]
GUCY2C	3rd	CD28 4-1BB	RV	T84	5:1; 10:1	% C (10:1): ~65%. ↑ Release of IFN- γ, TNF-α and IL-2.	[48]
HER2	2nd	4-1BB	LV	HCT116	0.3:1; 3:1; 9:1; 27:1	% C (1:9): ~50%. ↑ Release of IFN-γ, TNF-α, IL-2 and granzyme B.	[78]
MSLN	3rd	CD28 4-1BB	LV	HCT116	2.5:1	Complete elimination of MSLN^+^ tumour cells (~0 of normalised cell index). ↑ Release of IFN-γ and TNF-α.	[82]
NKG2DL	3rd	CD28 4-1BB	Minic. DNA	HCT116, LS174T	5:1; 10:1; 20:1	CAR-T cells significantly reduce the target cells (% C (10:1): ~30% HCT116 and 25% LS174) and also produce significant amounts of IFN-γ and IL-2.	[75]
PLAP	2nd	CD28	LV	LoVo, Caco-2, LS123	10:1	High cytotoxic effect and ↑ release of IFN-γ. Combination with α-PD-1, α-PD-L1 or α-LAG3 significantly increased C (% C: LoVo cells (CAR-T: ~65%; CAR-T + α-PD-1: 70%; CAR-T + α-LAG3: 80%); LS123 cells (CAR-T: ~65%; CAR-T + α-PD-1: 75%; CAR-T + α-PD-L1: 80%) and IFN-γ release.	[64]
TAG-72 CD30/TAG-72	2nd	CD28	RV	LS-C	1:5; 1:3; 1:2.5; 1:1.2	CD30/TAG-72-CAR-T cells show significantly higher C (% C (1:1.2): ~70%) in comparison with TAG-72-CAR-T cells	[77]

C: cytotoxicity; Co-st.: co-stimulatory domain; Gen.: CAR generation; LV: lentivirus; Minic.: minicircle; MSC: mesenchymal stem cell; Ref.: reference/s; RV: ɣ-retrovirus; ~: around.

**Table 2:** In vivo studies of CAR-T cells using mouse models of human colorectal cancer.

Target	Gen.	Co-st.	Mouse Model	CAR-T Cell Treatment	Efficacy	Safety	Ref.
CEA	2nd	4-1BB	HT29-RFP xenografts (female BALB/c nude mice)	5 × 10^6^ and 1 × 10^7^ cells (IV/2ds) +/− rhIL-12	Tumour reduction (day 21) and elimination (day 28) when combined with rhIL-12. ↑ Serum IL-2, IFN-γ and TNF-α.	No obvious body weight loss.	[73]
	2nd	CD28	LS174T xenografts (NSG mice)	2 × 10^6^ cells with 4 × 10^5^ IL7/IL12-expressing MSCs (SC/1d)	Improved tumour suppression and prolonged survival after combined treatment with CAR-T cells and IL7/IL12-expressing MSCs, co-inoculated with the tumour cells.	NR	[79]
	2nd	CD28	LS174 xenografts (Rag2^−/−^cɣ^−/−^ mice)	5 × 10^6^ cells (SC/1d)	Significant inhibition of tumour formation after CAR-T cell treatment co-inoculated with the target tumour cells.	NR	[77]
EGFRvIII	3rd	CD28 4-1BB	DLD-1 or miR-153-overexpressing DLD-1 xenografts (NSG mice)	2 × 10^6^ cells (IV/1d)	CAR-T cells eradicated the tumour in 3/5 DLD-1 xenografts and in 5/5 miR-153-overexpressing DLD-1 xenografts.	Little weight change.	[54]
EpCAM	3rd	CD28 4-1BB	HT29 or SW480 xenografts (female NOD/SCID BALB/c mice)	2 × 10^7^ cells (SC/1d)	Delay in tumour formation after CAR-T cell treatment co-inoculated with the target tumour cells.	No GvHD and no toxic changes in main organs.	[74]
	3rd	CD28 4-1BB	HRT-18G xenografts (NSG mice)	1 × 10^7^ cells (IP/8ds)	Transient (mRNA) CAR-T cells significantly increase survival of late-stage CRC mouse models.	NR	[72]
GUCY2C	3rd	CD284-1BB	CT26.hGUCY2C syngeneic mouse model (BALB/c mice) and T84. FLuc xenografts (NSG mice)	1 × 10^7^ murine CD8^+^ CAR-T cells (IP/1d)	Murine CAR-T cells induced tumour reduction in both mouse models.	No intestinal toxicity due to cross-reactions.	[48]
HER2	2nd	4-1BB	PDX model (SCID-NPG mice)	2 × 10^6^ cells (IV/1d)	Complete tumour eradication after 2 months and elimination of tumour re-inoculation. ↑Persistence (16% of CD3^+^ cells are CAR-T cells at day 28).	NR	[78]
**MSLN**	3rd	CD28 4-1BB	HCT116 xenografts (NCG mice) and PDX model (PDX-col0092 mice)	2.5 × 10^6^ cells (IV/1d)	Xenograft model: tumour reduction and durable response (until the endpoint); persistence (7.5% of CD3^+^ cells are CAR-T cells at day 10). PDX model: reduction in large (1000 mm^3^) and small (50 mm^3^) tumours; MSLN-CAR detected in serum at endpoint.	No significant body weight changes. GvHD: significant hair loss after 120 days in 1/5 PDX mice.	[82]
**NKG2DL**	3rd	CD28 4-1BB	HCT116-Luc xenografts (male NOD/SCID mice)	1 × 10^7^ CD8^+^ CAR-T cells (IV/2ds)	Tumour growth suppression and persistence (NKG2D-CAR detected in the tumour sections after 25 days).	Gradual loss of body weight. No toxicity in healthy tissues.	[75]
	1st	-	HCT116-Luc xenografts (NGS mice)	1x10^7^ CAR-γδ T cells (IP/6ds)	Transient (mRNA) CAR-γδ T cells delayed tumour growth, but tumours regrowth after treatment.	NR	[87]
**PLAP**	2nd	CD28	LoVo xenografts (male NSG mice)	1 × 10^7^ cells (IV/3ds)	Decrease in tumour growth and persistence (CAR-T cells detected in blood after 16 days).	No decrease body weight and no changes in serum AST, ALT and amylase.	[64]

ALT: alanine aminotransferase; AST: aspartate aminotransferase; Co-st.: co-stimulatory domain; d(s): dose(s); Gen.: CAR generation; GvHD: graft-versus-host disease; IP: intraperitoneal; IV: intravenous; NCG: NOD CRISPR Prkdc IL2Rɣ; NSG: NOD/SCID/IL-2Rγc^null^; NOD: non-obese diabetic; NR: not reported; PDX: patient-derived xenograft; Ref.: reference; SC: subcutaneous; SCID: severe combined immune deficiency.

## 5. Clinical Trials of CAR-T Cells for CRC Patients

In the last few years, the growing application of cell immunotherapy in multicentre clinical trials has provided clinical evidence that CAR-T cells are a promising option for cancer treatment. Currently, there are some CAR-T therapies approved in the United States (FDA) and Europe (EMA) for commercialization and they are directed against haematological tumours. However, CAR-T therapy for solid tumours is a step behind due to the difficulties they present. In this group of cancers, the most developed therapy (phase IIb of clinical development) is directed against CEA-positive pancreatic adenocarcinoma with metastasis to the liver (NCT04037241). In this clinical trial, a CAR-T targeting the CEA antigen is infused into the liver and studied for efficacy and safety in combination with systemic chemotherapy versus only chemotherapy. On the other hand, a clinical trial in phase I/II has been finished for EGFRvIII-positive glioma, which consists of a non-myeloablative lymphodepleting preparative regimen of cyclophosphamide and fludarabine in combination with the EGFRvIII-CAR-T cells and aldesleukin (IL-2) (NCT01454596). In the phase I trial of this study, the administration of only the EGFRvIII-CAR-T cells did not show a clinically significant effect in patients with glioblastoma multiforme (median PFS = 1.3 months; median OS = 6.9 months), although tumour regression had been observed in mouse models [88,89].

With regard to CRC, several clinical trials have been approved against a wide variety of antitumour antigens. In Table 3, we summarise the information reported on the clinicaltrials.gov website related to CAR-T cell therapies against CRC. According to these data, most of the trials are in the early stages, i.e., phase I or phase I/II; therefore, their main study objectives are safety, dose level and maximum tolerable dose. The most frequently studied targets in CAR-T cell therapy for CRC are CEA and NKG2DL, followed by EGFR and HER2. The first CAR-T clinical trial for CRC, which started in 2014, studied the safety and efficacy of second-generation CEA-CAR-T cells in patients with CRC, and also included lung, gastric, breast and pancreatic cancer patients (NCT02349724). After receiving the CAR-T cells, 7 out of 10 patients who had progressive disease (PD) with previous treatments were observed to have stable disease (SD), and two patients remained with SD for more than 30 weeks. Furthermore, serum CEA levels decreased in most patients and two patients showed tumour shrinkage. Regarding safety, fever was considered the most significant event related to the infusion of CAR-T cells. Two out of the ten treated patients experienced high fever (39–40 °C) within a few hours after CAR-T cell treatment and the fever reappeared several times within 1–4 days after cell infusion. This symptom was treated with nonsteroidal anti-inflammatory drugs (NSAIDs) as needed [44].

The same type of anti-CEA CAR-T cells has been studied in CEA-positive adenocarcinoma with liver metastases patients (NCT02416466 and NCT02850536 trials), administrated by hepatic artery infusions to improve the delivery of the CAR-T cells to liver metastases. Moreover, direct trans-arterial hepatic infusion contributes to avoid the risk of cytokine release syndrome (CRS), a common potential life-threatening complication derived from the systemic administration of CAR-T cells. In the NCT02416466 trial, different adverse events were observed in all patients; however, none of them experienced serious adverse events associated with CAR-T cell treatment [90]. The second trial included a pressure-enabled drug delivery system, a device designed to overcome high intra-tumour pressures, enhancing the CAR-T cell delivery to the liver metastases 5.2-fold. Within this trial, the authors reported the treatment of one patient and showed an increase in OS and a complete metabolic response. As in the previous trial, CAR-T infusion was not related to any serious adverse events graded higher than 3 or associated with on-target/off-tumour effects. Other relevant adverse events (grade 3) included dehydration, fever, hyperglycaemia, hypertension, hypokalaemia, hyponatraemia and hypophosphataemia [91].

Regarding the type of CAR, the second generation is the most frequently assayed, CD28 usually being the co-stimulatory domain, and 4-1BB in some other cases. A third-generation CAR that combined CD28 and 4-1BB co-stimulatory domains was used in one clinical trial. The fourth generation, also known as armoured CAR-T cells or TRUCKs, was also applied in two clinical trials. One of them studied an EGFR-directed CAR-T cell with inducible production and the release of transgenic IL-12 cytokine (NCT03542799), which is capable of modulating the tumour microenvironment and attracting and activating innate immune cells to attack the tumour [36]. The other fourth-generation CAR is an anti-MSLN CAR-T that includes the inducible production and secretion of anti-PD-1 nanobodies (NCT04503980). Since PD-1 is a negative regulatory receptor expressed by immune cells and stimulated by PD-L1, which is usually expressed by tumour cells, anti-PD-1 nanoantibodies can avoid the inhibiting interaction, thus increasing the immune cell response [92]. Besides PD-1, CTLA-4, lymphocyte-activation gene 3 (LAG3) and T cell membrane protein 3 (TIM3) are also immune checkpoint molecules whose interaction with their cognate ligands results in the suppression of immune cell functions. Since they are upregulated in the tumour microenvironment, it is worth including mechanisms to inhibit these interactions when designing next-generation CARs [93].

An atypical CAR, consisting of the NKG2D extracellular domain as the ligand-binding region and endogenous DAP10 as the co-stimulatory domain, has been used in three different studies. The clinical trials have not reported efficacy results yet, but the researchers of the NCT03018405 trial indicated that there is not toxicity up to 3 × 10^9^ CAR-T cells per dose [94].

There are only two allogeneic CAR-T cell therapies against CRC in clinical trials (NCT04107142 and NCT03692429), which have recently started, in 2019 and 2020, respectively. Both CAR-T cell therapies are directed against the NKG2D ligands but differ in the strategy to make them suitable for allogeneic use. The first one uses γδ T cells specifically as a source to manufacture the allogeneic CAR-T cells [72], since their TCR activation is not MHC haplotype restricted, so they are unlikely to induce GvHD. This subtype of T cells represents 1–5% of circulating lymphocytes, but they are predominant in some epithelial tissues. Indeed, their tissue residency patterns make them more accessible to non-inflamed tumours, and they are also able to recognise a huge variety of tumour antigens through their innate cytotoxicity receptors, reducing the immune escape [95,96]. The second CAR-T cell product uses a non-gene-edited strategy based on a TCR inhibitory molecule (TIM) sequence included in the construct. Therefore, when the peptide is expressed, it interferes with endogenous TCR signalling and controls GvHD [97]. In this clinical trial, seven patients remained with SD for at least 3 months after treatment, and no GvHD or grade 3 adverse events related to the treatment were observed [97].

**Table 3:** Clinical trials of CAR-T cells in patients with colorectal cancer.

Target	Gen.	Co-st.	Use	CRC Subtype	ID	Ph.	n	CAR-T Cell Treatment	Results	Adverse Events	Status	Ref.
CD133	1st	-	Au.	CRC	NCT02541370	I/II	20	0.5–2 × 10^6^ cells/kg (2 ds)	NA for CRC patients (only reported for HCC patients).	NA for CRC patients (only reported for HCC patients).	C	[98]
CEA	2nd	CD28	Au.	CEA^+^ Liver met.	NCT02416466	I	8	1 × 10^10^ cells/d (3 ds) with IL-2 followed by SIRT	(*n* = 6) 67% PD and 33% SD in the liver, and 17% ND and 83% PD in the extrahepatic.	G 3 (*n* = 6): 33% colitis, 33% fever and 38% reduction in K^+^. No CRS or neurotoxicity.	C	[90]
	2nd	CD28	Au.	CRC	NCT02349724	I	75	5 DL: 10^5^–10^8^ CAR^+^ cells/kg (split d: 10%, 30% and 60% per day)	(*n* = 10) 70% SD, 20% PD and 10% NE.	G 2 (*n* = 10): 20% fever (CAR-T related). G 3/4: lymphodepletion related. Minor CRS after high doses of CAR-T cells.	unk	[44]
	2nd	CD28	Au.	CEA^+^ Liver met.	NCT02850536	I	5	1 × 10^10^ cells/d (3 ds) with IL-2	(*n* = 1) Complete metabolic response within the liver over 13 months.	G 3 (*n* = 1): dehydration, fever, hyperglycaemia, hypertension, hypokalaemia, hyponatraemia, and hypophosphataemia. No on-target/off-tumour SAEs.	ANR	[91]
	NA	NA	Au.	mCRC	NCT02959151	I/II	20	1.25–4 × 10^7^ CAR^+^ T cells/cm^3^ tumour bulk (1 d)	NA	NA	unk	NA
	NA	NA	Au.	CRC	NCT03682744	I	18	NA	NA	NA	ANR	NA
	NA	NA	Au.	CRC	NCT04348643	I/II	40	NA	NA	NA	R	NA
	NA	NA	Au.	Stage III Liver met.	NCT04513431	eI	18	NA	NA	NA	NyR	NA
C-Met	NA	NA	Au.	CRC	NCT03638206	I/II	73	NA	NA	NA	R	NA
EGFR	3rd	CD28 4-1BB	Au.	EGFR^+^ mCRC	NCT03152435	I/II	20	NA	NA	NA	unk	NA
	4th	CD28 4-1BB	Au.	mCRC	NCT03542799	I	20	NA	NA	NA	unk	NA
EpCAM	2nd	CD28	Au.	Colon Cancer	NCT03013712	I/II	60	1–10 × 10^6^ CAR^+^ T cells/kg (1 d)	NA	NA	unk	NA
HER2	NA	NA	Au.	CRC	NCT02713984	I/II	0	NA	Reformed CAR structure due to safety considerations.	NA	W	NA
	NA	NA	Au.	CRC	NCT03740256	I	45	7 DL: 1–100 × 10^6^ cells (1 d) and oncolytic adenovirus CadVEC	NA	NA	R	NA
MSLN	4th	NA	Au.	CRC	NCT04503980	eI	10	4 DL: 1 × 10^5^–3 × 10^6^ cells/kg (1 d)	NA	NA	R	NA
MUC1	NA	NA	Au.	CRC	NCT02617134	I/II	20	NA	NA	NA	unk	NA
NKG2DL	NKR-2	End. DAP10	Au.	Liver met.	NCT03310008	I	36	3 DL: 10^8^–10^9^ cells/d (3 ds) and FOLFOX	NA	NA	ANR	[99]
	NKR-2	End. DAP10	Au.	Liver met.	NCT03370198	I	1	3 DL: 3 × 10^8^–3 × 10^9^ cells/d (3 ds)	NA	NA	ANR	[100]
	1st	-	All.	Unresec. mCRC	NCT03692429	I	49	3 DL: 1 × 10^8^–1 × 10^9^ cells/d (3 ds) and FOLFOX	Refractory unresec. mCRC (*n* = 15): 13% PR, 60% SD and 27% PD.	No treatment-related G ≥ 3 adverse events or GvHD.	R	[97]
	NKR-2	End. DAP10	Au.	CRC	NCT03018405	I	146	3 DL: 1–3 × 10^9^ cells/d (3 ds)	NA	No dose-limiting toxicity.	unk	[94]
	NA	-	All.	CRC	NCT04107142	I	10	3 DL: 3 × 10^8^–3 × 10^9^ CAR-γδ T cells/d (4 ds)	NA	NA	unk	NA
	2nd	4-1BB	Au.	Colon Cancer	NCT04270461	I	0	1–10 × 10^6^ cells/kg	Study withdrawn because of administrative reasons.	NA	W	NA
	2nd	4-1BB	Au.	CRC	NCT04550663	I	10	NA	NA	NA	NyR	[101]
PSMA	2nd	CD28	Au.	CRC	NCT04633148	I	35	UniCAR02-T cells with recombinant antibody derivative TMpPSMA	NA	NA	R	[102]

ANR: active, not recruiting; Au.: autologous; All.: allogeneic; C: completed; CRC: colorectal cancer; CRS: cytokine release syndrome; d(s): dose(s); DL: dose levels; eI: early phase I; End.: endogenous; G: grade of toxicities; Gen.: CAR generation; GvHD: graft-versus-host disease; HCC: hepatocellular carcinoma; mCRC: metastatic CRC; M: median; n: patient number; met.: metastasis; NA: not available; NE: not evaluable; ND: not detectable; NyR: not yet recruiting; Ph.: phase; PD: progressive disease; PR: partial response; R: recruiting; Ref.: reference; SD: stable disease; SIRT: selective internal radiation therapy; TIM: T-cell receptor inhibitory molecule; unk: unknown; Unresec.: unresectable; W: withdrawn.

## 6. Current Limitations and Future Perspectives

CAR-T cell immunotherapy has emerged as a very promising anticancer strategy, especially in the treatment of B-cell malignancies. In the context of solid tumours such as CRC, there are some obstacles and limitations that need to be addressed.

One of the most important aspects regarding CAR-T cell treatment of solid tumours is the need of CAR-T cells to infiltrate the tumour mass, since the tumour core is usually poorly irrigated [37] and there are biological barriers to be overcome by the cells, such as the tumour endothelium, which overexpresses receptors and ligands [103]. Colorectal cancer active TILs are known to express high levels of G-protein-coupled receptors, CXCR3 and CCR5, indicating their relevance in T-cell trafficking. Moreover, heparanase release has been associated with T cell antitumour activity in vivo in solid tumours. CAR-T cells might be designed to express CXCR3 and CCR5 in the membrane and to induce heparanase release, thus mimicking the behaviour of TILs and improving tumour infiltration. An alternative strategy consists of the design of CAR-T cells against molecules overexpressed in the newly formed blood vessels due to tumour angiogenesis, such as αvβ6 integrin and VEGF-2, thereby restricting nutrient supply and limiting the metastatic capacity of colorectal cancer [37].

The immunosuppressive tumour microenvironment that characterises solid tumours is another important limitation for CAR-T cell therapy success. The high level of hypoxia [37], low concentration of nutrients such as tryptophan necessary for T cell survival, proliferation and activation [54], as well as the high release of acid products derived from the accelerated metabolism of the tumour cells, contribute to generating a hostile environment that prevents the cytotoxic action of CAR-T cells against the tumour [37]. Additionally, some molecules such as PEG2 and adenosine, secreted in high amounts by transformed cells and tumour-associated macrophages, are capable of inhibiting T-cell proliferation [37]. Additionally, Tregs, myeloid-derived suppressor cells (MDSC) and tumour-associated macrophages and neutrophils contribute to immune evasion of the tumour by secreting TGF-β, IL-10, nitric acid and indoleamine dioxygenase 2–3 [37,104]. In an attempt to overcome these challenges, T cells expressing a dominant negative TGF-β receptor have been engineered [105]. Another proposed strategy is the use of chimeric receptors that transform the immune suppressive signals into stimulatory signals by combining the binding domain of the receptor of immunosuppressive factors such as IL-4 with the intracellular domain of IL-2 or IL-7 receptors. Additionally, T cells Redirected for Universal Cytokine Killing (TRUCKs) capable of inducing IL-12 release to counteract the immunosuppressive microenvironment and recruit the patient’s innate immune system have been developed [37].

However, the large majority of authors agree that combination therapy of CAR-T cells together with monoclonal antibodies (e.g., anti-PD1, anti-CTLA-4) or other therapeutic agents targeting the immunosuppressive microenvironment would result in a synergistic action capable of improving the efficacy of treatments to overcome limitations in the management of solid tumours [106,107].

The targets currently used for anti-CRC CAR-T cell therapy are usually not exclusive for tumour cells, which sometimes leads to on-target off-tumour toxicities [37,42,54,77,108]. Since the ideal CAR-T cell target should be only expressed in the tumour cells and absent in healthy tissue, CRC TSAs are promising candidates that must be further explored to develop novel CAR-T cell therapies.

Moreover, in order to improve the development of cancer immunotherapies, more relatable preclinical models that resemble the human disease and immune system are needed, since it is crucial to determine what drives cancer immunity and the immunological relationships between the cancerous cells and the rest of the organism, as well as to understand how this varies depending on the cancer type and subtype, among other topics that still remain unclear [109].

The potency of CAR-T cells often goes hand in hand with toxicity, since CAR-T cell treatment has the potential to trigger an exacerbated immune response as a consequence of increased tumour components released into tissues following malignant cell destruction by CAR-T cells, and the exacerbated production of pro-inflammatory cytokines (on-target/on-tumour toxicity). This may lead to CRS, which is a major problem of CAR-T cell therapy, since it may lead to fatal patient outcomes. The risk of CRS can be minimised by the administration of tocilizumab, an anti-human IL-6 receptor monoclonal antibody originally approved for the treatment of rheumatologic diseases. Moreover, since the risk of CRS is apparently dependent on tumour burden, administration of standard antitumour therapy before CAR-T cell administration with the purpose of reducing tumour volume has been also recommended to avoid this life-threatening adverse effect [37,41,77,103]. Some cases of severe transient inflammatory colitis [77] and respiratory failure [103] have also been reported as a result of CAR-T cell activity. Additionally, cytotoxic action without off-target effects will depend on the presence of specific tumour antigens not shared with healthy tissues [37]. At the moment, different strategies are being investigated to find a balance between efficacy and safety, including the use of monoclonal antibodies targeting IL-6 as rescue therapy; spacing treatment by splitting doses; using therapy in the early stages of cancer where the tumour mass is lower; or developing new CAR structures such as inhibitory CARs (iCARs), which trigger inhibitory signals when bound to a specific antigen expressed only by normal tissues, bispecific CARs to increase tumour specificity or CAR-T cells with on/off switches to control therapy safety, among others [37]. Moreover, local administration, such as trans-arterial hepatic infusion for liver tumours and metastases, has been proposed as an effective approach to increase efficacy and minimise toxicity in the treatment of solid tumours, in combination with additional systemic therapies [90].

## 7. Conclusions

Among the different cell therapies studied against CRC, CAR-T cells represent one of the most promising approaches. Numerous preclinical and clinical studies are ongoing to evaluate the efficacy and safety of CAR-T cells directed to a wide variety of molecular targets overexpressed in CRC, although the clinical development is still in its infancy (phase I and I/II clinical trials). The combination of CAR-T cells with immune-stimulating factors such as pro-inflammatory interleukins or additional immunotherapeutic strategies directed to inhibit the immunosuppressive tumour microenvironment have resulted in improved efficacy against CRC. Fourth-generation and next-generation CAR designs (e.g., tandem CARs), focused on improving the efficacy and reducing the toxicity of these treatments, need to be further investigated. Moreover, allogeneic CAR-T cell therapy against CRC has been barely explored until now, despite it being essential to reduce treatment costs and to make it affordable for public health systems. Although further research is needed, preclinical and clinical data point to CAR-T cell therapy as a promising strategy for the treatment of CRC, even at advanced stages.
